# Channel and Power Allocation for Multi-Cell NOMA Using Multi-Agent Deep Reinforcement Learning and Unsupervised Learning

**DOI:** 10.3390/s25092733

**Published:** 2025-04-25

**Authors:** Ming Sun, Yihe Zhong, Xiaoou He, Jie Zhang

**Affiliations:** College of Computer and Control Engineering, Qiqihar University, Qiqihar 161006, China; 2023911328@qqhru.edu.cn (Y.Z.); 2023935758@qqhru.edu.cn (X.H.); 2023935754@qqhru.edu.cn (J.Z.)

**Keywords:** non-orthogonal multiple access (NOMA), channel allocation, power allocation, multi-agent deep reinforcement learning, unsupervised learning, attention mechanism

## Abstract

Among the 5G and anticipated 6G technologies, non-orthogonal multiple access (NOMA) has attracted considerable attention due to its notable advantages in data throughput. Nevertheless, it is challenging to find the near-optimal allocation of the channel and power resources to maximize the performance of the multi-cell NOMA system. In addition, due to the complex and dynamically changing wireless communication environment and the lack of the near-optimal labels, conventional supervised learning methods cannot be directly applied. To address these challenges, this paper proposes a framework of MDRL-UL that integrates the multi-agent deep reinforcement learning with the unsupervised learning to allocate the channel and power resources in a near-optimal manner. In the framework, a multi-agent deep reinforcement learning neural network (MDRLNN) is proposed for channel allocation, while an attention-based unsupervised learning neural network (ULNN) is proposed for power allocation. Furthermore, the joint action (JA) derived from the MDRLNN for channel allocation is used as a representation to be fed into the ULNN for power allocation. In order to maximize the energy efficiency of the multi-cell NOMA system, the expectation of the energy efficiency is used to train both the MDRLNN and the ULNN. Simulation results indicate that the proposed MDRL-UL can achieve higher energy efficiency and transmission rates than other algorithms.

## 1. Introduction

In the digital era, with the widespread use of smart devices and the continuous expansion of mobile internet services, global mobile data traffic has been growing explosively [1,2,3,4,5]. This trend has placed higher demands on existing wireless communication systems in terms of transmission rate. To meet these challenges, researchers have started to investigate new communication methods including 6G technologies [6]. In particular, non-orthogonal multiple access (NOMA) technology has been proposed and rapidly developed. It has become an effective means to improve the performance of wireless communication systems and is receiving significant attention in the research of 5G and future network technologies.

NOMA technology allows multiple users to transmit signals simultaneously in the same frequency band and time resource, which significantly improves spectral efficiency compared to the traditional orthogonal multiple access (OMA) technology [7,8,9]. The base station allocates different power levels to users on the same subchannel, enabling the receiver to decode signals of users one by one in order of their power levels using the successive interference cancelation (SIC) technique [10], thereby achieving multi-user sharing in the power domain. The introduction of SIC technology provides a new solution to the problem of allocating limited wireless communication resources, but it also presents a series of technical challenges.

To achieve efficient operation of NOMA technology, the key is to optimally allocate channel and power resources in a dynamic network environment. This joint channel and power allocation problem has been proven to be NP-hard [11,12]. Namely, to find the optimal solution in the current environment, all possible combinations of resource allocations must be traversed. However, in multi-user and multi-cell scenarios, even if this exhaustive search method is feasible, the consumption of time and computing resources is unbearable. According to the most relevant related works summarized in [13], most of the research objectives are mainly focused on the sum rate and energy efficiency. It can be seen that how to reasonably allocate these resources to maximize the sum rate and the energy efficiency has become an important issue in the technical research and practical applications of NOMA systems.

Many traditional algorithms have been proposed to solve the resource allocation problem [14,15,16,17,18,19,20,21], such as game theory methods and convex optimization techniques. However, due to the highly complex channel characteristics, these traditional methods may have limitations in dealing with the resource allocation problems under large-scale environments. Additionally, while these traditional resource allocation strategies are feasible to a certain extent, their efficiency and reliability often fail to meet the demands of modern wireless communication systems in dynamic network environments. In response to this, researchers have begun exploring the use of the artificial intelligence technologies [22,23,24,25,26,27,28,29,30,31,32,33] to solve the resource allocation problem.

Studies have shown that deep learning has unique advantages in addressing resource allocation problems in the field of wireless communications [31,32,33,34,35]. For example, in supervised deep learning, common approaches include genetic algorithms or other heuristic algorithms that generate labels to train neural network models for channel assignment and power control [34,35]. In deep reinforcement learning, researchers typically employ deep Q-network (DQN) to formulate channel allocation policies and use deep deterministic policy gradient (DDPG) algorithm for continuous power control in dynamically changing wireless environments [33]. In addition, the application of unsupervised learning to the power control problem has also attracted much attention. Through unsupervised learning, the system is able to autonomously learn and adjust the power allocation strategy based on the characteristics of the channels without explicitly labeled data. This approach not only reduces the dependence on manually labeled data, but also better adapts to the complex and changing wireless communication environment.

Inspired by the above, this paper divides the resource allocation problem of the multi-cell NOMA systems into two sub-problems of channel allocation and power allocation and uses the methods of deep reinforcement learning and unsupervised learning to solve them. The contributions of this paper are listed as follows.

First, this paper treats the channel allocation problem as a process of selecting users for channels. In each environmental state, the system allocates channels to corresponding users until all available channel resources are exhausted. Our goal is to find a near-optimal allocation scheme that maximizes the overall system performance. Due to the lack of near-optimal label data, conventional supervised learning methods cannot be directly applied to the channel allocation. Hence, this paper proposes a channel allocation method based on multi-agent deep reinforcement learning, motivated by the advantage of deep reinforcement learning in subchannel selection [36]. This method leverages the deep reinforcement learning and the collaborative characteristics among multiple agents to explore various channel allocation solutions and obtains a near-optimal channel allocation solution by training the neural network.

Second, since power control in NOMA systems is a continuous non-convex optimization problem and considers the timeliness and accuracy of the system, it is very difficult to obtain the near-optimal solution by using supervised learning or discretization methods. Therefore, this paper proposes a power allocation method based on unsupervised learning, motivated by the fact that unsupervised learning is more efficient and effective than reinforcement learning in solving continuous power optimizations [24]. Meanwhile, to better exploit the hidden information of the channel allocation schemes to improve solutions of power allocation, the proposed unsupervised network for power allocation adopts a transformer architecture. In order to reduce the training burden of the power allocation network, it only uses the attention mechanism to deeply analyze the channel allocation scheme. Note that unsupervised learning can mine hidden information without labeling, but traditional neural networks (e.g., CNN, RNN) are difficult to model complex dependencies. The attention mechanism explicitly quantifies the interactions among data points through QKV (query–key–value) dynamic weight assignment, and transforms the implicit correlations discovered by unsupervised learning into interpretable weight matrices. Meanwhile, since the unsupervised objective (e.g., energy efficiency maximization in this paper) is usually non-convex, traditional neural networks are prone to fall into local optimum. In addition, the attention mechanism using residual connection and layer normalization can stabilize the gradient flow of unsupervised training and combine with multi-head attention to explore multiple solution spaces in parallel and reduce the sensitivity of non-convex optimization, which has been proven to be able to achieve good performance with reasonable training time.

Finally, we have made comparisons of the proposed framework with the other approaches, such as the single-agent DQL (DRLA) and the multi-agent deep reinforcement learning (MDRLA) proposed [13], the DQN-DDPG dual-layer deep learning resource allocation framework [33], and the resource allocation algorithm based on the deep deterministic policy gradient and unsupervised learning (DDPG-UL) [32]. Simulation results indicate that our proposed framework achieves better system performance than these algorithms. Compared to DQN-DDPG and DDPG-UL, the framework proposed in this paper shows a higher upper bound and greater stability in a time-varying dynamic environment.

This paper is organized as follows. Section 2 provides a detailed description of the system model. Section 3 explains the problem formulation of resource allocation in NOMA systems. The channel allocation method based on the multi-agent reinforcement learning is proposed in Section 4, and the power allocation method based on the attention-based unsupervised learning is proposed in Section 5. Section 6 presents simulation comparisons between the proposed framework and other algorithms. Section 7 provides conclusions.

## 2. System Model

**Figure 1:**
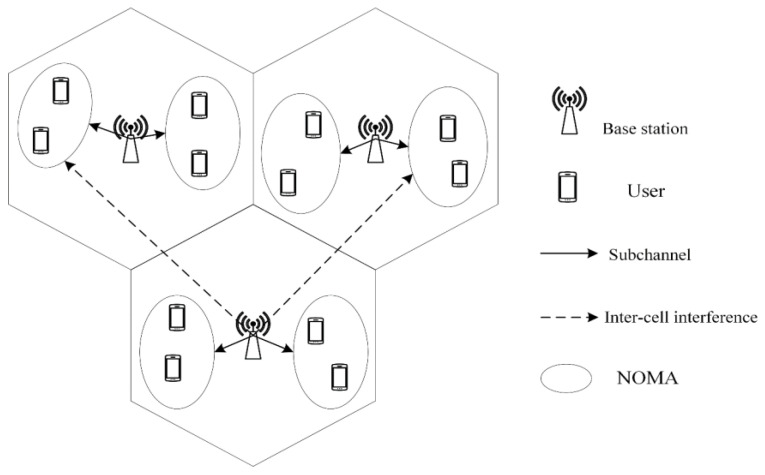
System model.

This paper considers a centralized downlink multi-cell NOMA system, as shown in Figure 1. Each cell contains a base station that transmits data to multiple users over wireless channels, and the signals of different users are superimposed through channel and power allocation in the system. At the receiver, users can recover their individual signals by using the serial interference cancelation (SIC) technique. It is assumed that the multi-cell NOMA system consists of *M* cells, *K* users, and *N* orthogonal subchannels. The total channel bandwidth is $B_{tot}$, so the bandwidth of each subchannel is $B_{tot}/N$. Concurrently, each subchannel in each cell can be allocated to ${k^{\prime }}_{n}$ users for multiplexing, where ${k^{\prime }}_{n}$ denotes the number of users for which subchannel *n* is multiplexed. Let $S_{m,u}^{n}$ denote the transmission signal of the user *u* on the subchannel *n* of the base station *m*. The superimposed signal $S_{m}^{n}$ on the subchannel *n* of the cell *m* can be expressed as follows:

(1) $$S_{m}^{n}=\sum_{u=1}^{K_{m}}G_{m,u}^{n}\sqrt{p_{m,u}^{n}}S_{m,u}^{n}$$

where $K_{m}$ is the number of users in cell *m*, $G\in R^{M\times N\times K}$ is the channel allocation indicator, and $G_{m,u}^{n}=1$ indicates that the base station *m* allocates the subchannel *n* to the user *u*, otherwise $G_{m,u}^{n}=0$, and $p_{m,u}^{n}$ is the power that the base station *m* allocates to the user *u* on the subchannel *n*.

When the superimposed signal is transmitted through the wireless channel to the receiver, the signal received by the user *u* of the cell *m* on the subchannel *n* is represented as follows:

(2) $$\begin{array}{l} j_{m,u}^{n}=\underset{\mathrm{Desired}\;\mathrm{signal}}{\underset{︸}{G_{m,u}^{n}H_{m,u}^{n}\sqrt{p_{m,u}^{n}}S_{m,u}^{n}}}+\underset{\mathrm{intra}-\mathrm{cell}\;\mathrm{interference}}{\underset{︸}{H_{m,u}^{n}\sum_{r=1,r\neq u}^{K_{m}}G_{m,r}^{n}\sqrt{p_{m,r}^{n}}S_{m,r}^{n}}}\\ \;\;\;\;\;\;\;\;\;+\underset{\mathrm{inter}-\mathrm{cell}\;\mathrm{interference}}{\underset{︸}{\sum_{c\in M,c\neq m}H_{c,u}^{n}\left(\sum_{\hat{u}=1}^{K_{c}}G_{c,\hat{u}}^{n}\sqrt{p_{c,\hat{u}}^{n}}S_{c,\hat{u}}^{n}\right)}}+\underset{\mathrm{AWGN}}{\underset{︸}{z_{m,u}^{n}}} \end{array}$$

where $H_{m,u}^{n}$ represents the channel gain between the base station m and the user u over the subchannel n. The superimposed signal consists of four components: the desired signal, the intra-cell interference, the inter-cell interference (ICI), and the additive white gaussian noise (AWGN) with zero mean and variance $\sigma_{n}^{2}$.

It is assumed that the user’s receiver in this multi-cell NOMA system is equipped with the SIC technique to decode the superimposed signals in the subchannel. The SIC technique is realized by using the channel-to-noise ratio (CNR) of the user u on the subchannel n with the base station *m*, i.e., $\psi_{m,u}^{n}$, as expressed below:

(3) $$\psi_{m,u}^{n}=\frac{G_{m,u}^{n}{\left|H_{m,u}^{n}\right|}^{2}}{\sigma_{n}^{2}+\sum_{c\in M,c\neq m}{\left|H_{c,u}^{n}\right|}^{2}p_{c}^{n}}$$

where $p_{c}^{n}$ is the sum of the power allocated by the base station *c* to the users on the subchannel *n*, i.e., $p_{c}^{n}=\sum_{\hat{u}=1}^{K_{c}}p_{c,\hat{u}}^{n}$. Then, the users in the same subchannel are ranked according to the magnitude of their CNRs. According to the fairness principle of the NOMA system, users with higher CNR are allocated to lower power, while users with lower CNR are allocated to higher power. Additionally, users allocated with higher power will regard the signals of users allocated with lower power as interference. Therefore, the SIC can decode the superimposed signal according to the powers of users. In this process, once the SIC decodes a user signal, interference from the signal is eliminated.

In this paper, each subchannel is assumed to be multiplexed by two users. It is assumed that the base station of the cell m allocates the subchannel n to two users, $u_{0}$ and $u_{1}$, where $\left|\psi_{m,u_{0}}^{n}\right|\geq \left|\psi_{m,u_{1}}^{n}\right|$, so that the user $u_{0}$ with the higher CNR can eliminate the interference from the user $u_{1}$ with the lower CNR on the subchannel n, i.e., $p_{m,u_{1}}^{n}\geq p_{m,u_{0}}^{n}$. Therefore, after SIC, the signal-to-noise ratios (SINRs) of the user $u_{0}$ and the user $u_{1}$ on the subchannel n can be written as

(4) $$\begin{array}{c} SINR_{m,u_{0}}^{n}=\frac{{\left|H_{m,u_{0}}^{n}\right|}^{2}p_{m,u_{0}}^{n}}{\sigma_{n}^{2}+\sum_{c\in M,c\neq m}{\left|H_{c,u_{0}}^{n}\right|}^{2}p_{c}^{n}}\\ SINR_{m,u_{1}}^{n}=\frac{{\left|H_{m,u_{1}}^{n}\right|}^{2}p_{m,u_{1}}^{n}}{\sigma_{n}^{2}+\sum_{c\in M,c\neq m}{\left|H_{c,u_{1}}^{n}\right|}^{2}p_{c}^{n}+{\left|H_{m,u_{1}}^{n}\right|}^{2}p_{m,u_{0}}^{n}} \end{array}$$

Then, the transmission rate provided by base station *m* for the user $u_{0}$ and the user $u_{1}$ on the subchannel *n* is formulated as follows:

(5) $$\begin{array}{l} R_{m,u_{0}}^{n}=\frac{B_{tot}}{N}\log_{2}(1+SINR_{m,u_{0}}^{n})\\ R_{m,u_{1}}^{n}=\frac{B_{tot}}{N}\log_{2}(1+SINR_{m,u_{1}}^{n}) \end{array}$$

The sum rate and the energy efficiency of this NOMA system can be formulated as follows:

(6) $$\begin{array}{c} R_{\mathrm{sum}}=\sum_{m=1}^{M}\sum_{n=1}^{N}\sum_{u=1}^{K_{m}}R_{m,u}^{n}\\ E_{\mathrm{sum}}=\frac{R_{\mathrm{sum}}}{\sum_{m=1}^{M}\sum_{n=1}^{N}\sum_{u=1}^{K_{m}}p_{m,u}^{n}+p_{0}} \end{array}$$

where $p_{0}$ is the inherent loss of the network base station equipment.

## 3. Problem Formulation and Resource Allocation

Allocating the limited power and channel resources for each cell to maximize the overall performance of the NOMA system is a significant challenge in NOMA research and is proven to be an NP-hard problem [11,12]. This paper takes the energy efficiency as the optimization objective for the multi-cell NOMA system. Specifically, the constrained optimization problem for resource allocation in this multi-cell NOMA system is formulated as follows:

(7) $$\begin{array}{l} \left(P1):\underset{G,P}{\max}E_{H}[E_{sum}\right]\\ \;\;\;\;\;\;\;\;\;\;\;\;s.t.\;\;\;C_{1}:\sum_{u=1}^{K_{m}}G_{m,u}^{n}=2,G_{m,u}^{n}\in \left\{0,1\right\}\\ \;\;\;\;\;\;\;\;\;\;\;\;\;\;\;\;\;\;\;\;\;\;C_{2}:p_{m}^{\max}\geq p_{tot,m}\geq 0\\ \;\;\;\;\;\;\;\;\;\;\;\;\;\;\;\;\;\;\;\;\;\;C_{3}:p_{m}^{n}\geq 0,p_{tot,m}=\sum_{n=1}^{N}p_{m}^{n}\\ \;\;\;\;\;\;\;\;\;\;\;\;\;\;\;\;\;\;\;\;\;\;C_{4}:p_{m,u}^{n}\geq 0,p_{m}^{n}=\sum_{u=1}^{K_{m}}p_{m,u}^{n} \end{array}$$

In (6), *G* and *P* are the allocation schemes of channel and power; the $C_{1}$ constraint indicates that the base station m allocates the channel n to two users; the $C_{2}$ constraint indicates that the total transmission power $p_{tot,m}$ of the base station m does not exceed the maximum allowable power $p_{m}^{\max}$; the $C_{3}$ constraint indicates that the power of the base station m on the subchannel *n* $p_{m}^{n}$ is non-negative and the power of the base station is the sum of the subchannel powers; the $C_{4}$ constraint indicates that the power $p_{m,u}^{n}$ allocated by the base station *m* to the user *u* on the subchannel *n* is non-negative, and the sum of $p_{m,u}^{n}$ is equal to the power of the subchannel *n* $p_{m}^{n}$.

For the above constrained optimization problem P1, we need to find the near-optimal channel and power allocation schemes to maximize the energy efficiency of the NOMA system. Assuming that there are $K_{m}$ users in the cell *m*, then the NOMA system has $\prod_{m=1}^{M}{\left(C_{K_{m}}^{2}\right)}^{N}$ channel allocation schemes. This implies that the number of channel allocation schemes increases exponentially with the number of users and channels. Thus, it is extremely difficult to find the near-optimal channel allocation scheme by traditional methods. Moreover, since the channel gain is time-varying, it imposes the requirement of the timeliness of the resource allocation. Taking into account the above considerations, this paper proposes a resource allocation framework based on the multi-agent deep reinforcement learning and the unsupervised learning for the downlink multi-cell NOMA system.

## 4. Channel Allocation Based on Multi-Agent Deep Reinforcement Learning

In this section, we first treat the channel allocation problem as an independent optimization problem and propose the use of multi-agent deep reinforcement learning for the channel allocation problem. We then introduce the network model of the multi-agent deep reinforcement learning method and provide the corresponding training algorithm.

### 4.1. Channel Allocation Formulation

In this subsection, the constrained optimization problem P1 is decomposed into the following channel allocation problem P2, as shown below.

(8) $$\begin{array}{l} \left(P2):\underset{G}{\max}E_{H}[E_{sum}\right]\\ \;\;\;\;\;\;\;\;\;\;s.t.\;\;\;C_{1}:\sum_{u=1}^{K_{m}}G_{m,u}^{n}=2,G_{m,u}^{n}\in \left\{0,1\right\} \end{array}$$

In this paper, the goal of the problem P2 is to find a channel allocation scheme that maximizes the energy efficiency of the system. To achieve this goal, we treat the channel allocation problem P2 as a process of selecting users for subchannels. Since this process is inherently discrete and reinforcement learning has significant advantages in solving discrete problems, a multi-agent deep reinforcement learning approach is proposed to select appropriate users for each subchannel.

### 4.2. Channel Allocation Using Multi-Agent Deep Reinforcement Learning

**Figure 2:**
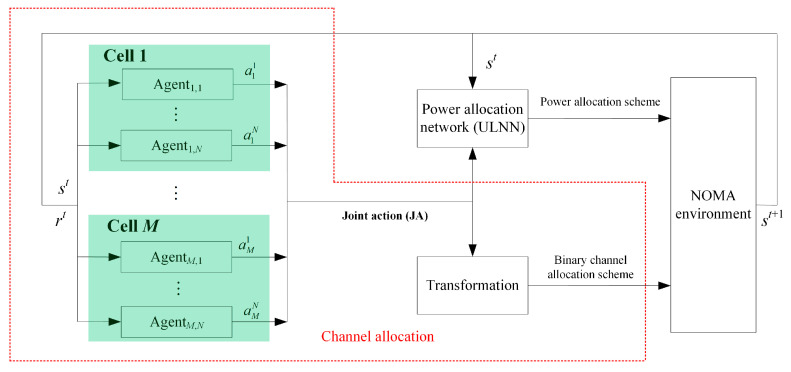
Schematic diagram of the multi-agent deep reinforcement learning for channel allocation aided by the power allocation.

In this subsection, the optimization constraint problem of channel allocation is modeled as a reinforcement learning task. Due to the large scale of the channel allocation scheme, using a single-agent structure for channel allocation may lead to system overload. Hence, we adopt a multi-agent structure, where each available subchannel in the base stations is regarded as an agent, and the downlink multi-cell NOMA system is viewed as the environment. Assume that there are M cells in the environment and the number of available subchannels in each cell is N; thus, the total number of agents in the environment is M×N. A schematic diagram of the multi-agent deep reinforcement learning for channel allocation aided by the power allocation is shown in Figure 2.

As shown in Figure 2, the agents in each cell first select an action $a_{m}^{n}$, respectively, from the action space $A_{m}$ based on the current state $s^{t}$ of the environment until all agents in the environment have completed their actions. Then, the actions of all agents are combined to form a joint action (JA). After that, both the JA and the global channel gain are fed into the power allocation network (i.e., ULNN in Section 5) to obtain a power allocation scheme, and the JA is transformed into a binary channel allocation scheme. With the operation of the power allocation scheme and the binary channel allocation scheme on the downlink multi-cell NOMA environment, a reward $r^{t}$ is obtained, and the system transitions to the next state $s^{t+1}$.

For convenience, the multi-agent deep reinforcement learning for the channel allocation is denoted as MDRL-CA. State, action, and reward of the MDRL-CA are defined as follows:

(1) State: In this paper, the energy efficiency is taken as the optimization goal in the NOMA system, each agent should consider the global information of the environment when selecting actions. Therefore, this NOMA system adopts a centralized architecture, and the state of the environment is characterized by the global channel gain information $H^{t}$, $H^{t}=[H_{1,1}^{1,t},\cdots H_{1,K}^{1,t},\cdots ,H_{M,K}^{1,t},\cdots ,H_{M,K}^{N,t}]$.
(2) Action: Since a channel should be allocated to two different users, the action represents a combination of two different users. The size of the action space of the agent in the cell m is $|A_{m}|=C_{K_{m}}^{2}$. It is noted that, since the number of users in each cell may be different, the action space of the agents in different cells may also be different. If there are 3 users in the cell *m*, the size of the action space of the agent in the cell *m* is $|A_{m}|=C_{3}^{2}=3$, and the action space is shown as $A_{m}=\left\{0:(1,2),\;1:(1,3),\;2:(2,3)\right\}$, where action 0 indicates that the agent has selected the 1st and 2nd users, action 1 indicates that the agent has selected the 1st and 3rd users, and action 2 indicates that the agent has selected the 2nd and 3rd users.
(3) Reward: Since the NOMA system uses the energy efficiency as the optimization goal, this paper adopts the energy efficiency as the reward function.

### 4.3. Proposed Multi-Agent Deep Reinforcement Learning Neural Network

**Figure 3:**
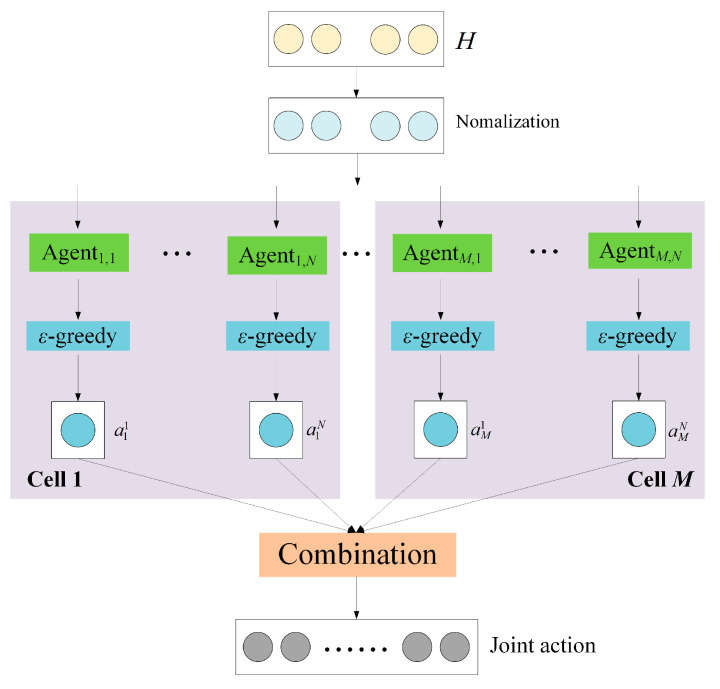
Proposed MDRLNN for channel allocation.

The structure of the proposed multi-agent deep reinforcement learning neural network (MDRLNN) is shown in Figure 3.

As shown in Figure 3, the proposed MDRLNN consists of *M* × *N* single-agent networks, each of which corresponds to a subchannel. This is because there are N available subchannels in each cell, and each cell contains N agents corresponding to the available subchannels.

When the global channel gain information is obtained from the environment, it is normalized first. The normalization h is shown in Equation (8).

(9) $$h^{t}=\frac{-\log_{10}({\bar{H}}^{t})-E\left[-\log_{10}({\bar{H}}^{t})\right]}{\sqrt{E\left\{{\left(-\log_{10}({\bar{H}}^{t})-E\left[-\log_{10}\left({\bar{H}}^{t}\right)\right]\right)}^{2}\right\}}}\;$$

In (8), both $h^{t}$ and ${\bar{H}}^{t}$ are vectors of M×N×K dimensions.

**Figure 4:**
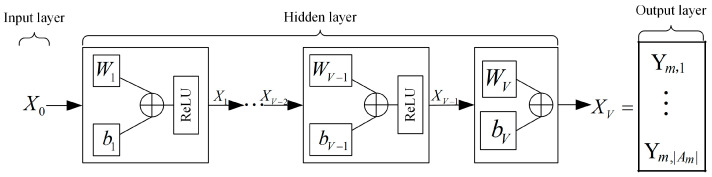
Single-agent neural network structure of agents in the cell *m*.

After the normalization, the system will distribute the information to the single-agent networks. It means that the single-agent networks have the same input dimension, determined by the size of the global channel gain. However, the output dimension of the single-agent networks is determined by the number of users in the cell. If there are $K_{m}$ users in the cell m, the single-agent network output dimension in the cell *m* is $\left|A_{m}\right|$, $|A_{m}|=C_{K_{m}}^{2}$, representing the number of combinations of two different user in the NOMA system. All of this implies that the N agents in the same cell have the same single-agent neural network structure. The single-agent neural network structure of agents of ${\mathrm{Agent}}_{m,1}$ to ${\mathrm{Agent}}_{m,N}$ in the cell m is shown in Figure 4.

Furthermore, as can be seen in Figure 3, each agent adopts an *ε*-greedy strategy to select actions. In this process, the base station retrieves user clusters and prioritizes allocating channels to those containing unassigned users, ensuring that all users obtain a subchannel.

Specifically, assuming there are $K_{m}$ users in cell *m*, the base station generates the user cluster index set $\left[A_{m,u_{1}},\cdots A_{m,u_{k_{m}}},\cdots A_{m,u_{K_{m}}}\right]$ based on the mapping function $A_{m}$, defined in Section 4.2. When $k_{m}=1$, $A_{m,u_{1}}=\left[0,1,\cdots ,K_{m}-2\right]$; when $k_{m}=K_{m}$, $A_{m,u_{K_{m}}}$ = $\left[K_{m}-2,\left(K_{m}-2\right)+\left(K_{m}-2\right),\cdots ,\left(K_{m}-2\right)+\cdots +1\right]$; and when $k_{m}\notin \left\{1,K_{m}\right\}$, $A_{m,u_{k_{m}}}$ is defined as follows.

(10) $$A_{m,u_{k_{m}}}=\left[\begin{array}{c} \left(k_{m}-1\right)-1,\\ K_{m}-1+\left(k_{m}-2\right)-1,\\ 2K_{m}-\left(1+2\right)+\left(k_{m}-3\right)-1,\\ \cdots ,\\ \left(k_{m}-1\right)K_{m}-\left(1+2+\cdots +\left(k_{m}-1\right)\right)\\ \cdots ,\\ \left(k_{m}-1\right)K_{m}-\left(1+2+\cdots +\left(k_{m}-1\right)\right)+\left(K_{m}-k_{m}\right)-1 \end{array}\right],\;\;\;k_{m}\notin \left\{1,K_{m}\right\}$$

During the agent’s action-selection process, the base station collects the cluster indices of users not yet assigned to any subchannel, shown as follows:

(11) $$A_{m}^{n}=\left[A_{m,u_{k_{m}}},\cdots ,A_{m,u_{{\tilde{k}}_{m}}}\right]$$

Based on this list, the optimal action $a_{m}^{n}$ will be selected. After all users are allocated to a subchannel, subsequent agents will select user clusters within the original action space $A_{m}$.

Finally, the actions selected by all agents are combined into a joint action $A^{t}$, as written below:

(12) $$A^{t}=\left[a_{1}^{1},\cdots ,a_{1}^{N},\cdots ,a_{m}^{n},\cdots ,a_{M}^{1},\cdots ,a_{M}^{N}\right]$$

where $a_{m}^{n}$ is the action of ${\mathrm{Agent}}_{m,n}$.

### 4.4. Training Algorithm for MDRLNN

A random search strategy is used to train the proposed MDRLNN to improve its performance. The detailed training steps are as follows:

First, an experience pool is established to store the sample data needed for training.

Second, an initial JA $A_{\mathrm{generate}}^{t}$ based on the current state $s^{t}$ of the environment is generated by the agents, and a batch of random JAs $\left\{A_{\mathrm{random},1}^{t},\cdots ,A_{\mathrm{random},B}^{t}\right\}$ are generated. Then, the JAs of $A_{\mathrm{generate}}^{t}$ and $\left\{A_{\mathrm{random},1}^{t},\cdots ,A_{\mathrm{random},B}^{t}\right\}$ are transformed into their corresponding channel allocation schemes $G_{\mathrm{generate}}^{t}$ and $\left\{G_{\mathrm{random},1}^{t},\cdots ,G_{\mathrm{random},B}^{t}\right\}$. In addition, the JAs of $A_{\mathrm{generate}}^{t}$ and $\left\{A_{\mathrm{random},1}^{t},\cdots ,A_{\mathrm{random},B}^{t}\right\}$ with the current state $s^{t}$ of the environment are, respectively, fed into the power allocation network (i.e., ULNN in Section 5) to obtain their corresponding power allocation schemes, $P_{\mathrm{generate}}^{t}$ and $\left\{P_{\mathrm{random},1}^{t},\cdots ,P_{\mathrm{random},B}^{t}\right\}$.

Third, $G_{best}^{t}=\arg\underset{G}{\max}E_{\mathrm{sum}}^{t}$ and $r^{t}=\max E_{\mathrm{sum}}^{t}$ are calculated, where

(13) $$E_{\mathrm{sum}}^{t}=\left\{E_{\mathrm{sum}}(H,G_{\mathrm{generate}}^{t},P_{\mathrm{generate}}^{t}),E_{\mathrm{sum}}(H,G_{\mathrm{random},1}^{t},P_{\mathrm{random},1}^{t}),\cdots ,E_{\mathrm{sum}}(H,G_{\mathrm{random},B}^{t},P_{\mathrm{random},B}^{t})\right\}$$

Finally, the sample data $\left(s^{t},A_{\mathrm{best}\;}^{t},G_{\mathrm{best}\;}^{t},r^{t}\right)$ is stored in the experience pool, where $A_{\mathrm{best}}^{t}$ is the JA corresponding to the channel allocation scheme $G_{\mathrm{best}}^{t}$.

The MDRLNN for the channel allocation is described in detail in Algorithm 1, shown as follows.
**Algorithm 1** MDRLNN for channel allocation**Input:** State space *S*; The experience pool; The initialized MDRLNN 
**Output:** The well-trained MDRLNN; joint action; channel allocation scheme
1: **for**
*t*
**do**
2:         **for** each cell *m* do
3:                 **for** each channel *n*
**do**
4:                        $a_{m}^{n}$ ← ε-greedy strategy based on the output of ${Agent}_{m,n}$
5:                 **end for**
6:         **end for**
7:         $G_{generate}^{t}$, $P_{generate}^{t}$ ← $A_{generate}^{t}$ ← [$a_{1}^{1}$, $a_{1}^{2}$, …, $a_{1}^{N}$, …, $a_{m}^{n}$, …, $a_{M}^{N}$]
8:         $G_{random,1}^{t}$, …, $G_{random,B}^{t}$}, {$P_{random,1}^{t}$,…,$P_{random,B}^{t}$} ← {$A_{random,1}^{t}$, …, $A_{random,B}^{t}$} ← randomly generated by the system
9:         $r^{t}$ = max$E_{sum}^{t}$
10:        $A_{best}^{t}$ ← $G_{best}^{t}$ ← argmax$E_{sum}^{t}$
11:        the sample data ($s^{t},A_{best}^{t},G_{best}^{t},\;r^{t}$) is stored in the experience pool
12:        **if** the number of the sample data in the experience pool reaches a certain level **then**
13:        A batch Ӽ of sample data ($s^{t},A_{best}^{t},G_{best}^{t},r^{t}$) is randomly selected
14:                 **for** each cell *j*
**do**
15:                        **for** each subchannel *i*
**do**
16:                                Loss(*θ*) = $\frac{1}{2Ӽ}\sum {\left(r^{t}-Y_{m,a_{j}^{i}}\right)}^{2}$
17:                                *θ* ← *Adam*(*θ*, $\nabla_{\theta }Loss(\theta )$)
18:                          **end for**
19:                 **end for**
20:                 test the MDRLNN
21:     **end if**
22: **end for**

## 5. Unsupervised Learning for Power Allocation

This section first formulates the power allocation problem and then presents an attention-based unsupervised learning neural network (ULNN) to solve the power allocation problem.

### 5.1. Power Allocation Formulation

In this paper, the constrained optimization problem P1 is decomposed into the following power allocation problem P3, as shown below:

(14) $$\begin{array}{l} \left(P3):\underset{P}{\max}E_{H}[E_{\mathrm{sum}}\right]\\ \;\;\;\;\;\;\;\;\;\;\;\;\;s.t.\;\;\;C_{1}:p_{m}^{n}\geq 0,p_{m}^{\max}\geq \sum_{n=1}^{N}p_{m}^{n}=p_{tot,m}\geq 0\\ \;\;\;\;\;\;\;\;\;\;\;\;\;\;\;\;\;\;\;\;\;\;\;C_{2}:p_{m,u}^{n}\geq 0,p_{m}^{n}=p_{m,u_{0}}^{n}+p_{m,u_{1}}^{n} \end{array}$$

where the constraint $C_{1}$ is formed by combining the constraints $C_{1}$ and $C_{2}$ in Problem P1, indicating that the sum of the channel powers $\sum_{n=1}^{N}p_{m}^{n}$ in cell *m* does not exceed the maximum allowable power of the base station $p_{m}^{\max}$. The goal of the problem is to find a power allocation scheme that maximizes the energy efficiency of the NOMA system. Considering the unique advantages of unsupervised learning for continuous optimization problems, this paper proposes an attention-based ULNN for power allocation in the downlink multi-cell NOMA systems.

### 5.2. Attention-Based ULNN for Power Allocation

In this subsection, we first introduce an attention-based ULNN to achieve channel power allocation. Then, the power of each subchannel is further allocated to the corresponding users.

**Figure 5:**
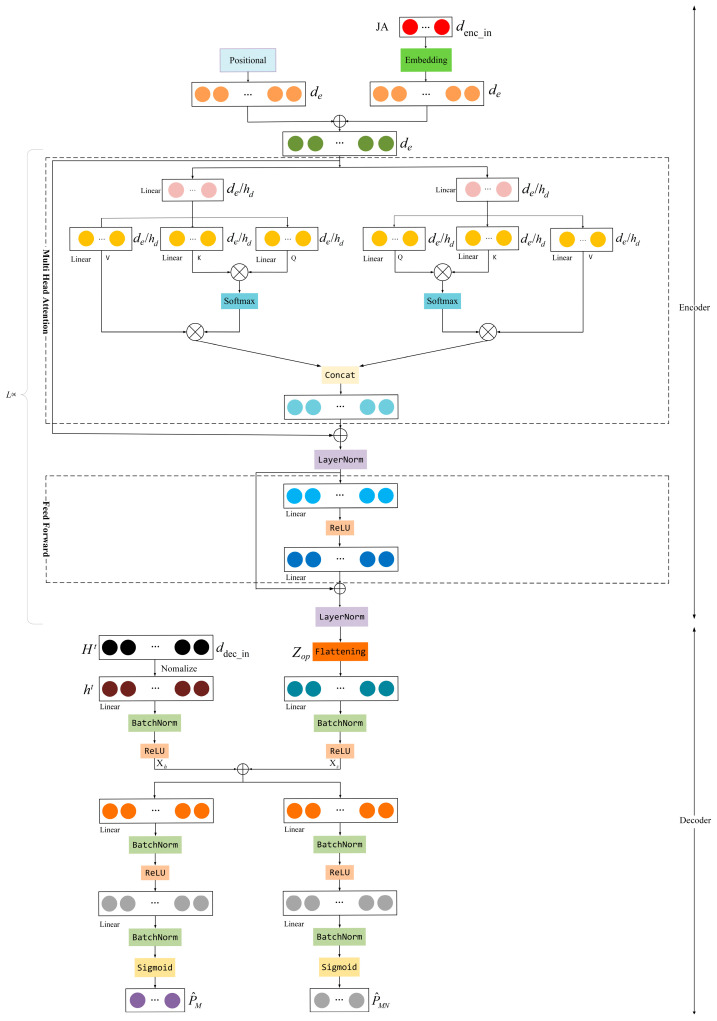
Structure of the proposed attention-based ULNN for power allocation.

Note that the JA obtained by the agents in the channel allocation can be viewed as the representations of the channel allocation scheme, and it contains more information than the binary channel allocation scheme. Based on this, the JA obtained by the agents in the channel allocation and the current state $s^{t}$ of the environment are fed into the proposed attention-based ULNN to obtain the near-optimal power allocation scheme P. Inspired by the transformer model [37], the encoder and decoder structures are used in the proposed ULNN, as shown in Figure 5.

(1) Encoder: At this layer, the critical information from the JA will be captured and preserved. Specifically, in the multi-cell NOMA system, the same action in a JA may correspond to different user clusters, since the number of users in each cell is not necessarily the same. To address this challenge, despite the relatively simple architecture of the traditional feedforward neural network, its fixed-dimensional input–output structure fails to effectively model long-range dependencies between sequence elements and lacks sensitivity to the input sequence order. In order to distinguish the semantic differences caused by positional variations, the encoder adds positional encoding *PE* to each action in the JA. Then, a multi-head attention mechanism is employed to capture interference relationships among user clusters, channel correlations, and other critical factors, thereby enhancing overall system performance. One attention head might focus on the power allocation of the subchannels within a cell, while the other attention heads are used to model the inter-cell interference. The specific process is shown as follows.

First, the JA output from the channel allocation is used as the input of the encoder, with the size of M×N. For each action in the JA, the encoder first linearly projects it into the initial $d_{e}$-dimensional output ($d_{e}$ is 512 in this paper). Since the same actions may represent the different combinations of users in different cells, the position encoding PE is added to this initial $d_{e}$-dimensional output to distinguish the actions, as shown below:

(15) $$\begin{array}{l} PE_{\left(pos,2i\right)}=\sin(pos/10000^{2i/d_{e}})\\ PE_{\left(pos,2i+1\right)}=\cos(pos/10000^{2i/d_{e}}) \end{array}$$

where *pos* is the position of the action in the JA.

Then, the initial output is encoded through *L* identical subsequent layers, where each layer consists of two sub-layers.

The first sub-layer is a multi-head attention layer, which contains 8 parallel attention heads $\left(h_{d}=8\right)$. The input of this layer is denoted by *X*, and is linearly projected to obtain the query (Q), the key (K), and the value (V), which can be expressed as

(16) $$\begin{array}{c} Q_{i}=XW_{i}^{Q}\\ K_{i}=XW_{i}^{K}\\ V_{i}=XW_{i}^{V} \end{array}$$

where $W_{i}^{Q}$, $W_{i}^{K}\in R^{d_{e}\times d_{k}}$, and $W_{i}^{V}\in R^{d_{e}\times d_{v}}$, $i=1,2,3,\cdots h_{d}$, and $d_{k}=d_{v}=d_{e}/h_{d}=64$.

Perform the attention operation on the result of each projection as follows:

(17) $$head_{i}=\mathrm{Attention}(Q_{i},K_{i},V_{i})=\mathrm{softmax}(\frac{Q_{i}K_{i}^{T}}{\sqrt{d_{k}}})V_{i}$$

The results are concatenated and then linearly transformed into a smaller dimensional output, as expressed below:

(18) $$MultiHead(Q,K,V)=Concat(head_{1},\cdots ,head_{h_{d}})W^{O}$$

where $W^{O}\in R^{h_{dv}\times d_{e}}$ and $h_{dv}=h_{d}\times h_{v}$. The second sublayer is the Feed-Forward Neural Network layer, which consists of two linear layers and an intermediate RELU activation layer. After each sublayer, residual connections and layer normalization operations are added sequentially.

(2) Decoder: The input of the decoder consists of two parts; one is the channel gain $H^{t}$ under the current environment, with the size of M×N×K, and the other is the output of the encoder, denoted as $Z_{op}$. First, the channel gain $H^{t}$ is normalized according to (8) and the result $h^{t}$ and the flattened $Z_{op}$ are each put into two separate channels, each consisting of one fully connected layer, one batch normalization layer, and one RELU activation layer. The outputs are written as follows:

(19) $$\begin{array}{l} X_{h}=\mathrm{RELU}(\mathrm{BN}(W_{h}h^{t}+b_{h}))\\ X_{z}=\mathrm{RELU}(\mathrm{BN}(W_{z}Z_{op}+b_{z})) \end{array}$$

where $W_{h}\in R^{d_{MNK}\times d_{NK}}$, $W_{z}\in R^{d_{MNe}\times d_{NK}}$, and $b_{h}$, $b_{z}\in R^{d_{NK}}$, $d_{MNK}=M\times N\times K$, $d_{MNe}=M\times N\times d_{e}$, and $d_{NK}=N\times K$. Therefore, both $X_{h}$ and $X_{z}$ are vectors of dimension N×K. These vectors are combined and passed through two fully connected hidden layers again; the output of the first fully connected hidden layer is

(20) $${\hat{P}}_{M}=\mathrm{sigmoid}(\mathrm{BN}({W_{{\hat{P}}_{M}}}^{\prime }\mathrm{RELU}(\mathrm{BN}(W_{{\hat{P}}_{M}}(X_{h}+X_{z})+b_{{\hat{P}}_{M}}))+{b_{{\hat{P}}_{M}}}^{\prime }))$$

where $W_{{\hat{P}}_{M}}\in R^{d_{NK}\times d_{MN}}$, ${W_{{\hat{P}}_{M}}}^{\prime }\in R^{d_{MN}\times M}$, $b_{{\hat{P}}_{M}}\in R^{d_{MN}}$, and ${b_{{\hat{P}}_{M}}}^{\prime }\in R^{M}$ and $d_{MN}=M\times N$. Therefore, there are M outputs of this fully connected hidden layer, as the power constraint variables of base stations, denoted as follows:

(21) $${\hat{P}}_{M}=[{\hat{p}}_{1}\cdots {\hat{p}}_{m}\cdots {\hat{p}}_{M}]$$

Next, based on the constrained optimization problem P3, ${\hat{P}}_{m}$ is performed as follows:

(22) $${\bar{p}}_{m}={\hat{p}}_{m}\times p_{m}^{\max}$$

where $p_{tot,m}$ = ${\bar{p}}_{m}$ denotes the total transmission power of the base station *m* and satisfies the $C_{1}$ constraints of P3. Then, the output of the second fully connected layer is the power constraint variables of subchannels, denoted as follows:

(23) $${\hat{P}}_{MN}=\mathrm{sigmoid}(\mathrm{BN}({W_{{\hat{P}}_{MN}}}^{\prime }\mathrm{RELU}(\mathrm{BN}(W_{{\hat{P}}_{MN}}(X_{h}+X_{z})+b_{{\hat{P}}_{MN}}))+{b_{{\hat{P}}_{MN}}}^{\prime }))$$

where $W_{{\hat{P}}_{MN}}\in R^{d_{NK}\times d_{MN}}$, ${W_{{\hat{P}}_{MN}}}^{\prime }\in R^{d_{MN}\times d_{MN}}$, $b_{{\hat{P}}_{MN}}=d_{MN}$ and ${b_{{\hat{P}}_{MN}}}^{\prime }=d_{MN}$. Thus, ${\hat{P}}_{MN}$ is an M×N dimensional vector, and can be denoted as

(24) $${\hat{P}}_{MN}=[{\hat{p}}_{1}^{1},\cdots ,{\hat{p}}_{1}^{N},\cdots ,{\hat{p}}_{m}^{n},\cdots ,{\hat{p}}_{M}^{1},\cdots ,{\hat{p}}_{M}^{N}]$$

In order to satisfy the $C_{2}$ constraint in P3, ${\hat{p}}_{m}^{n}$ is adjusted as follows:

(25) $$p_{m}^{n}=\frac{{\hat{p}}_{m}^{n}}{\sum_{n=1}^{N}{\hat{p}}_{m}^{n}}p_{tot,m}$$

where $p_{m}^{n}$ denotes the transmission power of base station m on subchannel n. Therefore, the constraint $C_{2}$ of problem P3 is satisfied.

To enforce the NOMA protocol [38] in NOMA systems (i.e., users with higher CNR receive lower power allocations to suppress inter-cluster interference), we formulate the user-level power allocation as an optimization problem under the constraints of P3. Specifically, assuming that two users ($u_{0}$,$u_{1}$) are multiplexed on subchannel *n* in cell *m* with CNR relationships $\left|\psi_{m,u_{0}}^{n}\right|\geq \left|\psi_{m,u_{1}}^{n}\right|$. In [8], the power allocation ratios ($a_{0}^{2}$,$a_{1}^{2}$) are established and satisfy $a_{0}^{2}+a_{1}^{2}$ = 1. By derivation, the following relationship is obtained:

(26) $$\frac{a_{0}^{2}}{a_{1}^{2}}\propto \frac{{\left|H_{m,u_{1}}^{n}\right|}^{2}}{{\left|H_{m,u_{0}}^{n}\right|}^{2}}$$

In [39], the power of each user is affected by the channel gain of that user as well as all users in that subchannel.

Combining the above, in this paper, the power ratio of users in each subchannel is set as follows:

(27) $$\frac{p_{m,u_{0}}^{n}}{p_{m,u_{1}}^{n}}=\frac{p_{m,u_{0}}^{n}}{p_{m}^{n}-p_{m,u_{0}}^{n}}=\frac{\psi_{m,u_{1}}^{n}}{\psi_{m,u_{0}}^{n}}$$

Then, the power of each subchannel is further allocated to the corresponding users, as shown below:

(28) $$\begin{array}{c} p_{m,u_{0}}^{n}=p_{m}^{n}\frac{\psi_{m,u_{1}}^{n}}{\psi_{m,u_{0}}^{n}+\psi_{m,u_{1}}^{n}}=\frac{{\hat{p}}_{m}^{n}\cdot \psi_{m,u_{1}}^{n}\cdot p_{tot,m}}{\sum_{n=1}^{N}{\hat{p}}_{m}^{n}\left(\psi_{m,u_{0}}^{n}+\psi_{m,u_{1}}^{n}\right)}\\ p_{m,u_{1}}^{n}=p_{m}^{n}-p_{m,u_{0}}^{n}=\frac{{\hat{p}}_{m}^{n}\cdot \psi_{m,u_{0}}^{n}\cdot p_{tot,m}}{\sum_{n=1}^{N}{\hat{p}}_{m}^{n}\left(\psi_{m,u_{0}}^{n}+\psi_{m,u_{1}}^{n}\right)} \end{array}$$

Note that the power $p_{m,k}^{n}=0$ if the subchannel *n* is not allocated to the user *k*. Finally, the optimal user power allocation scheme *P* with dimension M×N×K is obtained and can be expressed as follows:

(29) $$P=[p_{1,1}^{1},\cdots ,p_{1,K}^{1},\cdots ,p_{m,1}^{n},\cdots ,p_{m,k}^{n},\cdots ,p_{m,K}^{n},\cdots ,p_{M,1}^{N},\cdots p_{M,K}^{N}]$$

### 5.3. Training Algorithm for ULNN

To reduce the system load, the power allocation uses the same experience pool shared by the channel allocation. In the training process of the proposed attention-based ULNN, a batch of the channel gain information $H^{t}$ and the JA $A_{\mathrm{best}}^{t}$ sampled randomly from the experience pool are first fed into the proposed attention-based ULNN to obtain the power allocation scheme P. Next, the energy efficiency of the system is calculated based on the power allocation scheme P, the channel gain information $H^{t}$, and the channel allocation scheme $G_{\mathrm{best}}^{t}$. Finally, the negative expectation of the system energy efficiency is calculated as the loss function. To prevent overfitting during the training process, we additionally incorporate the *L*2 regularization into the loss function, it can be expressed as

(30) $$Loss=E_{G_{best}^{t},P}\left[-E+\lambda {\left\|w\right\|}_{2}^{2}\right]$$

where λ is the regularization parameter used to control the strength of regularization and ${\left\|w\right\|}_{2}^{2}$ is the sum of the squares of parameters in the weight vector of the proposed attention-based ULNN. The gradient of the loss function $\nabla_{\theta }Loss$ is calculated, and the parameters of the proposed attention-based ULNN are updated using the Adam optimizer.

The proposed attention-based ULNN for the power allocation is described in detail as Algorithm 2.
**Algorithm 2** ULNN for power allocation**Input:** State space *S*; The experience pool; The initialized ULNN 
**Output:** The well-trained ULNN; power allocation scheme
1: **for**
*t*
**do **
2:        **if** the number of the sample data in the experience pool reaches a certain level **then**
3:               $s^{t},A_{best}^{t},G_{best}^{t},\;r^{t}$ ← the sample data of the experience pool
4:               *P* ← output of the ULNN ← $s^{t}$, $A_{best}^{t}$
5:               $E_{sum}$ = $\sum_{1}^{M}\sum_{1}^{N}\sum_{1}^{K}\frac{\frac{B_{sum}}{N}\log_{2}\left(1+{SINR}_{m,k}^{n}\right)}{p_{m,k}^{n}+p_{0}}$
6:               *Loss* = $E_{G_{best}^{t},P}$[−$E_{sum}$ + λ${\left||w|\right|}_{2}^{2}$]
7:               *θ* ← *Adam*(*θ*, $\nabla_{\theta }Loss(\theta )$)
8:               Test the ULNN
9:        **end if**
10: **end for**

## 6. Simulation Results

In this section, simulations are used to evaluate the performance of the proposed resource allocation framework of MDRL-UL in time-varying dynamic environments by making comparisons with the DRLA [13], the MDRLA [13], the DDPG-UL [32], and the DQN-DDPG [33]. Additionally, we conduct the hyper-parameter analysis to show the impact of hyper-parameter on the proposed resource allocation framework during the testing process. Finally, we analyze the impact of the random search strategy on system performance.

### 6.1. Simulation Settings

In the simulation, we assume that all the base stations are located at the center of the cells, and the users are randomly distributed within 200 m around the base stations, where the number of cells is *M* = 3 and the number of users in each cell is $K_{m}$ = 4. The variance of the channel noise in the downlink multi-cell NOMA system is defined as $\sigma_{n}^{2}=B_{tot}N_{0}/N$, where $N_{0}=-170\mathrm{dBm}$ is the noise spectral density. The maximum transmission power of the base station is $p_{\max}=2∼12\mathrm{watt}$ and the subchannel bandwidth is $B_{n}=180\mathrm{kHz}$. The channel gain of the channel *n* between the user *k* and the base station *m* is represented as follows:

(31) $$H_{m,k}^{n}=10^{-({\mathrm{PL}}_{m,k}^{\upsilon }+\tau )/10}{\left|h_{m,k}^{n}\right|}^{2}$$

where ${\mathrm{PL}}_{m,k}^{\upsilon }$ denotes the path loss from the base station *m* to the user *k* with υ=3.2 being the path loss exponent; τ is the shadow fading, which is a normally distributed random variable with a mean of 0 and a standard deviation of 8; and $h_{m,k}^{n}$ represents the fast fading for the communication between base the station m and the user k on the channel n, which is an independent and identically distributed complex Gaussian random variable with zero mean and unit variance.

In the proposed framework of the MDRL-UL, the single-agent neural network in the MDRLNN is equipped with an input layer, three fully connected hidden layers, and an output layer. To balance system performance and complexity, the number of layers *L* in the encoder of the ULNN is set to 3, and the dimension of the position is set to 512. Based on this, the complexity of each method is shown in Table 1, where $T_{step}$ is the number of iterations, *L* is the number of layers, and $V_{i}$ is the number of neurons in layer *i*.

Before the experiments, 750 pieces of channel gain information were collected for training and testing the MDRLNN and the ULNN in the downlink multi-cell NOMA system. The learning rate for the MDRLNN is set to 0.0008, and the learning rate for the ULNN is set to 0.0001.

### 6.2. Performance Comparison

**Figure 6:**
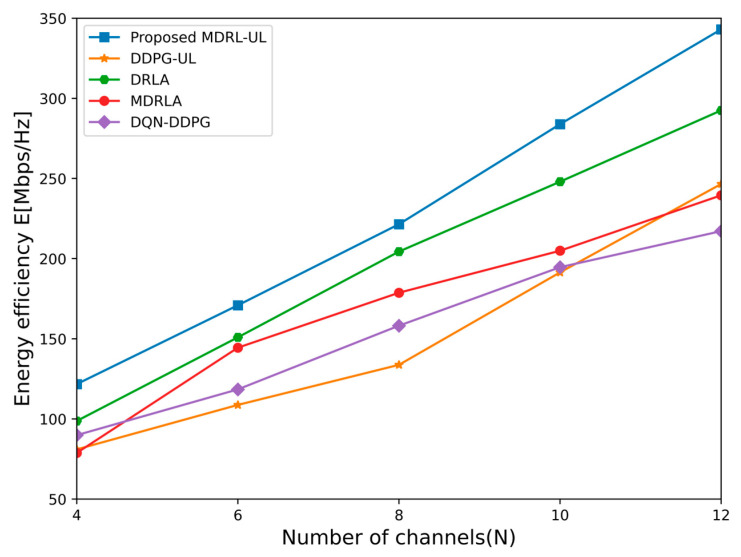
The energy efficiency obtained by different algorithms for different number of channels.

**Figure 7:**
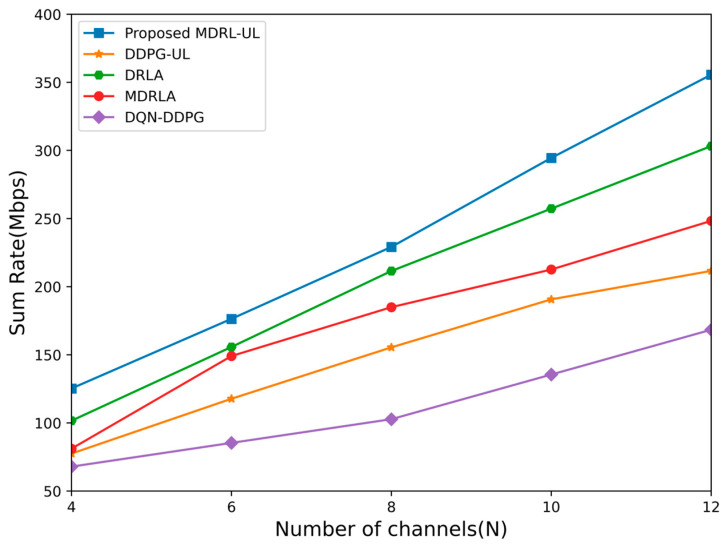
The sum rate obtained by different algorithms for different number of channels.

In the NOMA system, as the maximum transmission power of the base station is $p_{\max}=6W$, the energy efficiency and the sum rate of each algorithm for different number of channels are shown in Figure 6 and Figure 7. It can be seen that the energy efficiency and the sum rate achieved by the proposed framework of the MDRL-UL are higher than the four other algorithms. This is because the energy efficiency and the transmission rate are not only related to the quality of the channel but also affected by the interference from users in other cells. Each agent of the framework not only considers its own environmental information but also continuously collects its surrounding information through interactions among agents. However, since both MDRLA and DQN-DDPG adopt a distributed architecture where agents operate independently, they cannot guarantee the globally optimal solution. For the other two algorithms, DRLA and DDPG, although they exhibit lower complexity in Table 1, their reliance on a vast action space makes them highly sensitive to even minor deviations, drastically affecting system performance. In contrast, the proposed MDRL-UL method can find better allocation schemes of the channel and the power for the multi-cell NOMA system with lower complexity.

**Figure 8:**
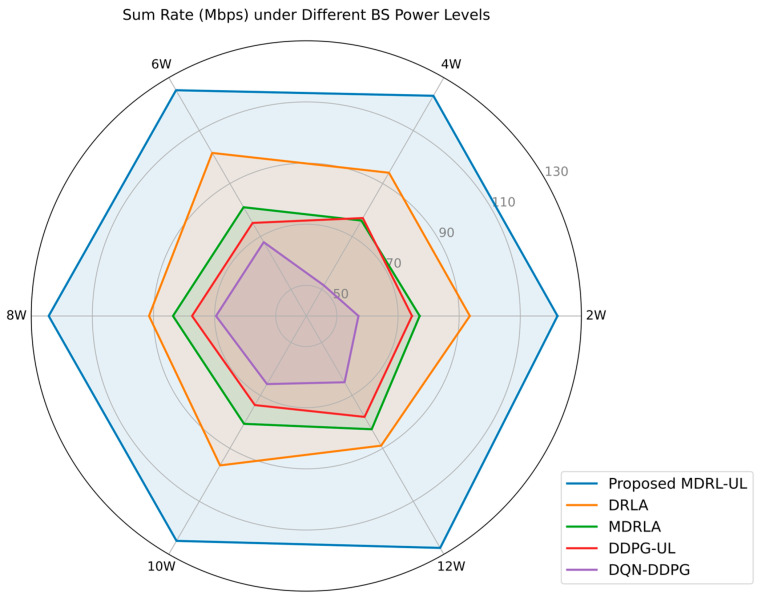
The sum rate obtained by different algorithms as the maximum transmission power of the base station varies from 2 W to 12 W.

Let the number of channels be fixed as *N* = 4. As the maximum transmission power of the base station varies from 2 W to 12 W, the sum rate obtained by different algorithms is shown in Figure 8. It can be seen from Figure 8, the sum rate of each algorithm fluctuates at a certain level. According to (3), (4), (5), and (6), as the maximum transmission power of the base station increases, the range of power that can be allocated to each user increases, and the interference from other cells also increases. Therefore, the sum rate of the NOMA system should remain stable. We can observe from Figure 8 that the sum rate obtained by the proposed framework of the MDRL-UL remains at 120 Mbps, with less fluctuation, indicating that the proposed framework of the MDRL-UL has higher stability and performance than other algorithms, which has higher stability and performance than other algorithms.

**Figure 9:**
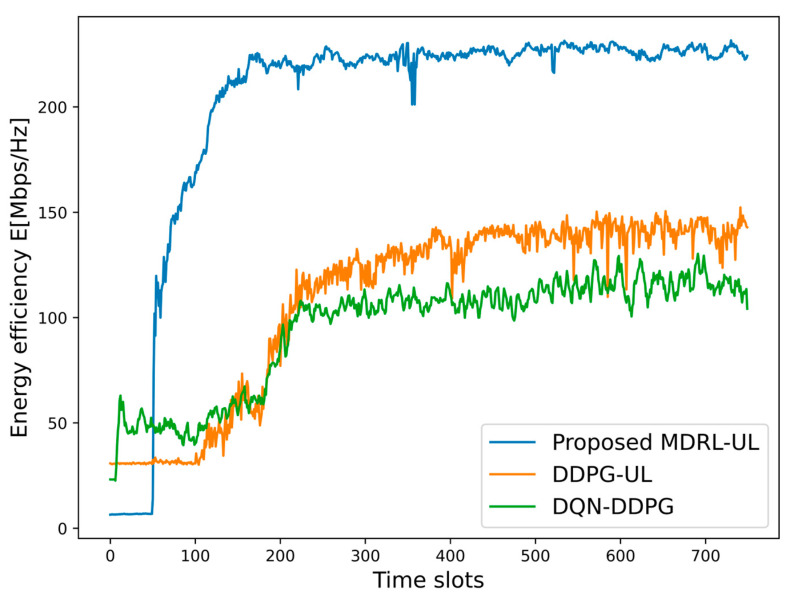
The energy efficiency of different algorithms in a time-varying dynamic environment.

**Figure 10:**
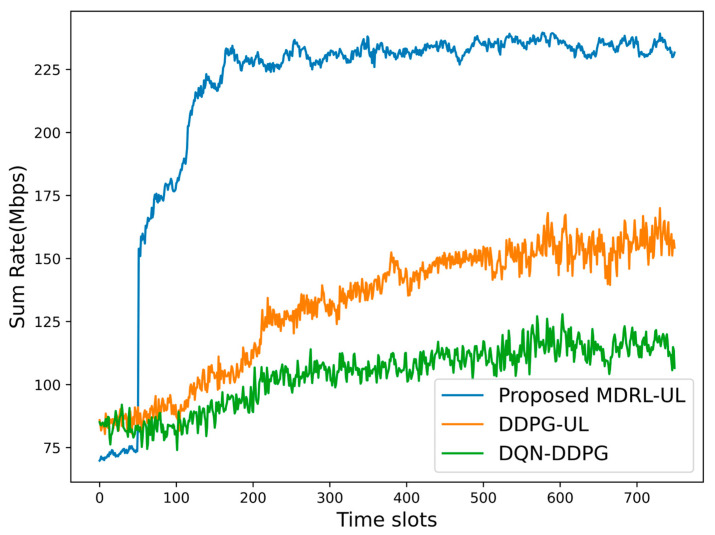
The sum rate of different algorithms in a time-varying dynamic environment.

Next, we compare the performance of the algorithms in a time-varying dynamic environment. Considering the time-varying nature of channel gains, the proposed frameworks of the MDRL-UL, the DDPG-UL, and the DQN-DDPG are to find the near-optimal power and channel allocation scheme at each time slot. In addition, the DRLA and the MDRLA are excluded from this comparison as they complete the resource allocation under a specific channel gain while not considering the time-varying nature of the channel gains. For the fairness of the comparison, the proposed frameworks of the MDRL-UL, the DDPG-UL, and the DQN-DDPG are applied to a time-varying dynamic environment with 3 cells, 8 channels, and 12 users. As time slots increase, the energy efficiency and the sum rate of different algorithms are shown in Figure 9 and Figure 10. According to the simulation results in Figure 9 and Figure 10, it can be found that the proposed framework of the MDRL-UL is more stable and has better performance than the DDPG-UL and the DQN-DDPG, which proves the effectiveness of the framework in the time-varying dynamic environment.

**Figure 11:**
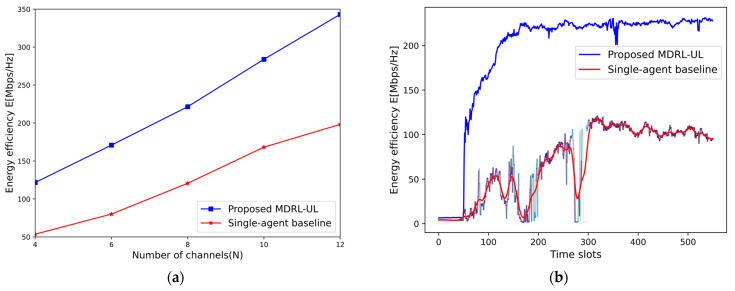
(**a**) the energy efficiency obtained by the proposed MDRL-UL algorithm and the single-agent baseline method under different number of channels. (**b**) the energy efficiency obtained by the proposed MDRL-UL algorithm and the single-agent baseline method in a time-varying dynamic environment.

In order to test the advantages of multi-agent reinforcement learning in NOMA systems, a single-agent baseline method is implemented to compare with the MDRL-UL method in terms of system performance, as shown in Figure 11. It can be seen that the energy efficiency of the proposed MDRL-UL framework outperforms the single-agent baseline method for all channel numbers, and the gap between the two increases as the channel number increases. In Figure 11b, it can be found that the NOMA system under multi-agent has a higher energy efficiency and is more stable. In addition, because the single-agent baseline method also needs to collect global channel gain information, its state space and action space will grow exponentially with the number of channels and users, thus increasing the overhead of resources. In the face of high-dimensional time-varying environments, the single agent requires a huge number of neural network parameters to capture deep information, which may cause an excessive or even unbearable load. For the multi-agent with complexity $O\left(T_{step}\cdot \left(N+1\right)\right)\cdot O\left(\sum_{i\in \left[1,L-1\right]}V_{i}V_{i+1}\right)$, it can reduce the number of parameters of each agent by cooperating with each other, thus reducing the computational load while maintaining the overall stability.

**Figure 12:**
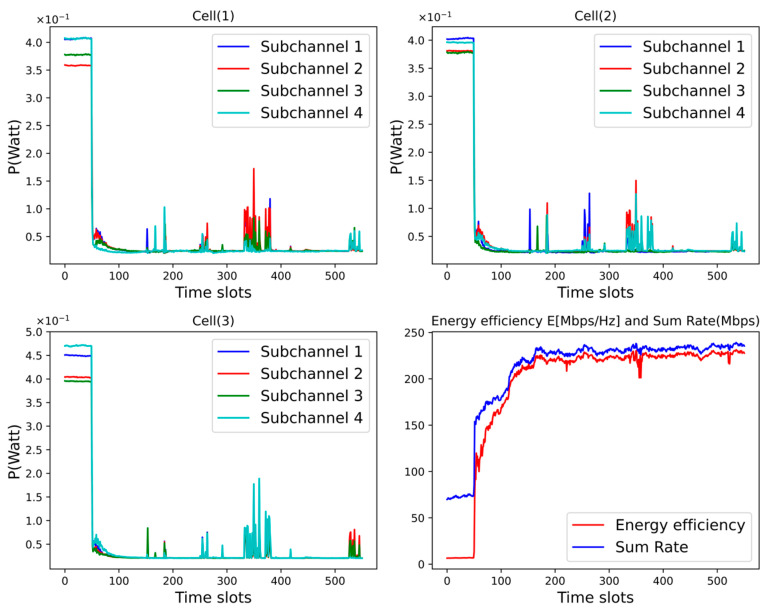
The impact of each subchannel power on system performance under unsupervised learning.

In addition, to verify the impact of unsupervised learning on the system performance, we plot the power of the user clusters corresponding to the subchannels in each cell and the system performance under each time slot, as shown in Figure 12. We can find that the power of each subchannel is maintained at a low level over time. This is because, according to (4), (5), and (6), as the power increases, each user cluster is similarly subject to higher external interference. Therefore, when the channel conditions are better, the unsupervised learning neural network allocates a lower power to each subchannel to reduce the external interference suffered by each user cluster, thus improving the system performance. Under the poor channel conditions, it can be found that the subchannel powers can adjust each other to ensure the overall stability of the system and maintain the system performance at a high level.

### 6.3. Hyper-Parameter Analysis

In this subsection, we will analyze the effect of the parameters on the proposed framework of the MDRL-UL. For comparison, we assume that there are M = 3 cells, N = 8 channels, and K = 12 users in the NOMA system.

**Figure 13:**
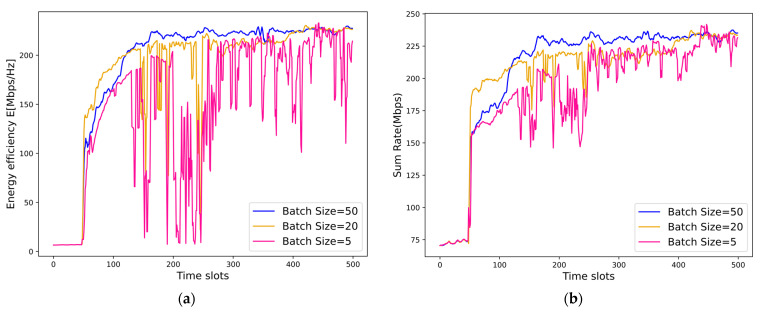
(**a**) Convergence of the energy efficiency of the proposed MDRL-UL framework under different batch sizes. (**b**) Convergence of the sum rate of the proposed MDRL-UL framework under different batch sizes.

The impact of the batch size is demonstrated in Figure 13. We can observe that the energy efficiency and the sum rate of the NOMA system increase with the batch size. This is because, with a larger batch size, the proposed framework of the MDRL-UL can explore more resource allocation schemes, which helps to discover deeper relationships and obtain a better resource allocation scheme. We can also observe that as the batch size increases, the process of convergence becomes more stable, and the upper bounds are higher.

**Figure 14:**
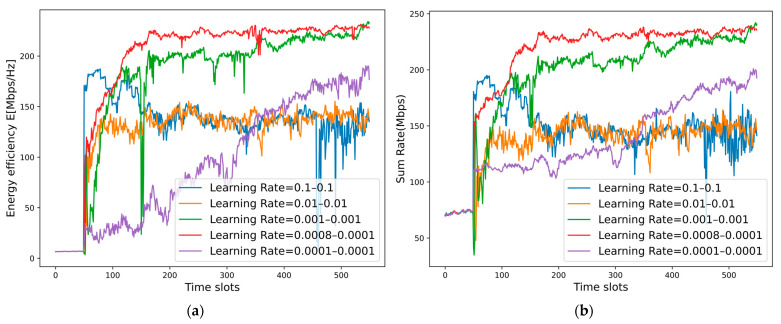
(**a**) Convergence of the energy efficiency of the MDRL-UL framework with different learning rate. (**b**) Convergence of the sum rate of the proposed MDRL-UL framework with different learning rate.

Figure 14 shows the effect of the learning rate, where the batch size is set to 50. When the learning rate of MDRLNN and ULNN are both set to 0.1 or 0.01, we can see that the energy efficiency and the sum rate both stabilize at a low level, indicating that the NOMA system cannot obtain a good resource allocation scheme at each time slot. When the learning rate of MDRLNN and ULNN are both set to 0.001, the results converge to a higher level, but the process is not stable enough. If the learning rate is set even lower, e.g., 0.0001, the energy efficiency and the sum rate will continue to improve, but the convergence speed will be too slow. Considering these observations, we set the learning rate of MDRLNN to 0.0008 and the learning rate of ULNN to 0.0001. It can be seen that both the energy efficiency and the sum rate have converged to a higher level, and the testing process is more stable.

### 6.4. Impact of the Random Search Strategy

**Figure 15:**
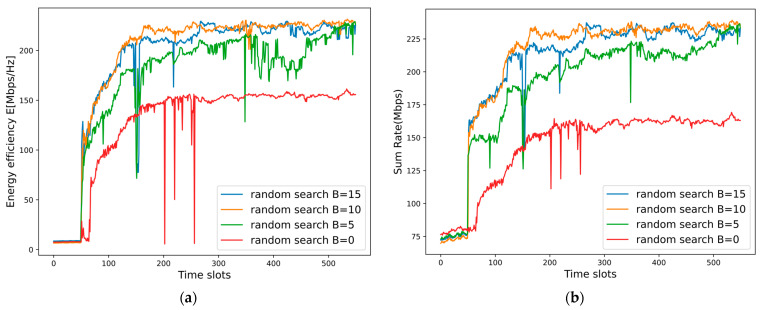
(**a**) Impact of the random search strategy on the energy efficiency. (**b**) Impact of the random search strategy on the sum rate.

In this paper, the impact of the random search strategy on system performance is shown in Figure 15, where the batch size is set to 50, and the learning rates of the MDRLNN and ULNN are set to 0.0008 and 0.0001. With the random search strategy, we can see that the energy efficiency and the sum rate of the NOMA system are significantly improved. This is due to the fact that the random search strategy enables the system to explore a greater number of channel allocation schemes per time slot, which prevents the neural networks of the proposed MDRL-UL algorithm from becoming trapped in local optima.

However, when the number *B* of randomly generated JAs by the NOMA system is set lower, such as 5, the system convergence slows down because the MDRLNN needs more time slots to train to find a better channel allocation scheme. Also, when *B* is set larger, e.g., as 15, we find that although the system performance is able to converge to a better level in a shorter period, there is still a large oscillation in the system performance. This is due to the over-reliance on the results of the random search strategy during the training process, which leads to the phenomenon of overfitting. When the system environment, i.e., channel gain $H_{m,u}^{n}$, changes drastically, the neural network is unable to adapt to the change in time. And when *B* is set to 10, it can be found that the energy efficiency and the sum rate can converge to a higher value in a shorter time and remain stable. This is because, based on the result of the random search strategy, the system can provide a high-quality action label for the neural network in the early stage of reinforcement learning training to avoid blind exploration of the agents and accelerate convergence.

As a result, this demonstrates that the random search strategy, combined with a reasonable number *B*, is able to improve the system performance by finding a better channel allocation scheme for the NOMA system in a time-varying dynamic environment.

## 7. Conclusions

To maximize the performance of the NOMA system, we propose a resource allocation framework of MDRL-UL that combines deep reinforcement learning and unsupervised learning. The framework treats the resource allocation as a constrained optimization problem and divides it into two sub-problems of channel allocation and power allocation. To resolve the sub-problem of channel allocation, a multi-agent deep reinforcement learning approach is introduced, where each agent corresponds to a subchannel in a cell and can allocate the subchannel to two users based on the global channel gain information. When all the channels are allocated, a channel allocation scheme is obtained. With the channel allocation scheme, an unsupervised learning approach using an attention-based neural network is proposed to obtain a power allocation scheme. Simulation results show that the proposed framework of MDRL-UL outperforms other algorithms in terms of energy efficiency and sum rate, and the testing process is more stable than other algorithms, even if the environment is time-varying. In addition, the random search strategy can significantly improve the testing performance of the NOMA system.

## Figures and Tables

**Table 1:** Complexity of each method.

Method	Complexity
MDRL-UL(Proposed)	$O\left(T_{step}\cdot \left(N+1\right)\right)\cdot O\left(\sum_{i\in \left[1,L-1\right]}V_{i}V_{i+1}\right)$
DRLA	$O\left(T_{step}\cdot M\right)\cdot O\left(\sum_{i\in \left[1,L-1\right]}V_{i}V_{i+1}\right)+T_{step}$
MDRLA	$O(T_{step})\cdot O\left(\sum_{i\in \left[1,L-1\right]}V_{i}V_{i+1}\right)+T_{step}$
DDPG-UL	$O(2T_{step})\cdot O\left(\sum_{i\in \left[1,L-1\right]}V_{i}V_{i+1}\right)$
DQN-DDPG	$O(T_{step}\cdot 2N)\cdot O\left(\sum_{i\in \left[1,L-1\right]}V_{i}V_{i+1}\right)$

## Data Availability

As part of the multi-cell NOMA system, the global channel gains in this paper are publicly available in the figshare repository: https://doi.org/10.6084/m9.figshare.26893618.
